# Proximal Row Carpectomy With Soft Tissue Interposition: A Systematic Review of Clinical Outcomes

**DOI:** 10.1177/15589447231221245

**Published:** 2024-01-30

**Authors:** Neveadan Aravinthan, Salwa Siddiqui, Moin Khan, Jaydeep Moro, Paula A. Pino, Carlos Prada

**Affiliations:** 1 McMaster University, Hamilton, ON, Canada; 2 Pontificia Universidad Católica de Chile, Santiago, Chile

**Keywords:** wrist, arthritis, proximal row carpectomy, scaphoid, wrist surgery, arthroplasty, interposition arthroplasty

## Abstract

Proximal row carpectomy (PRC) with soft tissue interposition arthroplasty (STIA) presents an alternative approach to addressing wrist arthritis patterns involving the capitate and/or lunate fossa, in lieu of wrist arthrodesis. This systematic review aimed to evaluate clinical outcomes and techniques associated with PRC-STIA in patients with advanced wrist arthritis. We conducted a systematic review using databases including PubMed, Embase, MEDLINE, and the Cochrane Central Register of Controlled Trials. Inclusion criteria involved articles reporting outcomes of patients who underwent PRC-STIA with at least 1 relevant outcome. The analysis encompassed 8 studies involving 106 patients (108 wrists) meeting the inclusion criteria. A majority of patients were men (69%, n = 88), with a mean age of 54.4 ± 12.7 years and an average follow-up of 4.8 ± 6.3 years. Dorsal capsule was the most commonly interposed tissue (63%, 5 out of 8 studies). Patients receiving STIA achieved comparable patient-reported outcome measures scores to those undergoing PRC alone. Postoperative pain, measured by the Visual Analog Scale, averaged 3.7 ± 0.6. The Disabilities of the Arm, Shoulder, and Hand score averaged 27.8 ± 8, while the Patient-Rated Wrist Evaluation score averaged 41.5 ± 25.9. Five complications were reported in three studies. The addition of STIA into PRC for patients with capitate and/or lunate fossa cartilage degeneration yielded outcomes akin to traditional PRC, improving wrist function, pain, and grip strength in a safe and straightforward manner. Future research should prioritize high-quality comparative studies, extended follow-up periods, and standardized core outcome measures for a more comprehensive understanding of its role in wrist arthritis treatment.

## Introduction

Proximal row carpectomy (PRC) is a motion-preserving surgical technique, first described by Stamm^1^ in 1944, to treat patients with advanced degenerative changes in the wrist. This surgery involves the excision of the bones of the entire proximal carpal row (scaphoid, lunate, and triquetrum), allowing the proximal pole of the capitate to articulate with the lunate fossa of the radius.^2^ This salvage procedure is currently recommended for patients experiencing wrist pain due to conditions such as: (1) scaphoid nonunion advanced collapse; (2) scapholunate advanced collapse; (3) Kienböck’s disease; and (4) long-term or unstable perilunate dislocations.^3^ Consequently, meeting the traditional eligibility criteria for this procedure mandates the integrity of both the chondral surfaces of the lunate fossa of the radius and the capitate proximal pole, as the procedure’s success hinges on the integrity of these surfaces.

Surgical options for the treatment of wrist arthritis can be divided into motion-preserving surgeries and total wrist arthrodesis (TWA). Total wrist arthroplasties also preserve motion but are only used in a selected group of patients because of their short durability and high complication rate.^4^

The main limitation of PRC and four corner fusion (4-CF) surgeries is that both require a healthy lunate fossa for the newly created radiocarpal joint to function pain-free. This restricts their use in advanced stages of carpal collapse, as most arthritic conditions have a predictable wear pattern involving the surfaces of the lunate fossa, the proximal pole of the capitate, and/or the proximal surface of the lunate. The 4-CF technique, in addition, is relatively contraindicated in patients with low bone healing potential (eg, smokers) or a deteriorated overall health condition.^1^ Traditionally, under these circumstances, TWA and total wrist arthroplasty are the chosen treatment options.

However, following the initial description by Salomon and Eaton^5^ in 1996, patients with degenerative changes of the proximal pole of the capitate and/or the lunate fossa of the radius started to be treated with a modification of the classic PRC technique which added an interposition arthroplasty. The role of the soft tissue interposition (STI) is to decrease the contact pressures between the worn articular surfaces, potentially reducing pain, while preserving motion and avoiding a TWA. Furthermore, it is believed that the soft tissue interposition arthroplasty (STIA) also improves grip strength, decreases joint contact pressures, minimizes patients’ pain, and could potentially improve the procedure durability.^2,4^ In fact, 2-year follow-up data suggest that PRC with STIA has better results of active range of motion (ROM) and grip strength for the treatment of wrist arthritis compared with PRC only.^6^

Despite the initial satisfactory outcomes shown by a small group of studies, this treatment option is still not widely used and other surgical alternatives, such as the capitate resurfacing arthroplasty, have gained popularity. The purpose of this systematic review was to evaluate the outcomes of patients with wrist arthritis involving the lunate fossa and/or the capitate proximal pole treated with PRC with STIA and to review the different types of STI used.

## Methods

This systematic review was conducted according to the methods outlined in the Cochrane Handbook for Systematic Reviews of Interventions and reported according to the Preferred Reporting Items for Systematic Reviews and Meta-Analyses (PRISMA) statements.^7^

### Search Strategy

PubMed, Embase, MEDLINE, and CENTRAL databases were searched from the date of inception to March 10, 2021. The search strategy was refined with a senior author (C.P.) to identify all studies involving PRC with any type of STIA and their outcomes (Appendix). In addition, Medical Subject Headings and Emtree terms were used in different combinations and supplemented with free text to increase search sensitivity. These search terms were also entered onto Google Scholar and ClinicalTrials.gov, to ensure no relevant articles were excluded. Two reviewers (N.A., S.S.) completed a review of the articles independently and conflicts were resolved in the following stage of the screening protocol.

### Eligibility Criteria

A systematic screening approach in accordance with PRISMA guidelines (Supplemental Figure 1) was conducted to filter for relevant articles and performed through Covidence (www.covidence.org, Veritas Health Innovation Ltd). Articles assessing outcomes of populations undergoing PRC + STIA for wrist arthritis either as the only group or as a subgroup within the study population which met the inclusion criteria were included.

Inclusion criteria were: (1) studies evaluating outcomes of patients undergoing PRC with STIA; (2) at least 1 outcome reported and stratified for population of interest; (3) human studies; and (4) all languages. The exclusion criteria were: (1) review articles; (2) technique articles without outcomes reported; (3) cadaver/nonhuman/biomechanical studies; and (4) full-text article not available.

### Study Screening

The study screening was performed in duplicate by 2 independent reviewers (N.A., S.S.) following in accordance with PRISMA and Revised Assessment of Multiple Systematic Reviews from title to full-text screening stages.^7,8^ The reviewers performed searching, perusal, inclusion and exclusion of relevant studies, and quality assessments. Discrepancies during the screening stage were resolved through a senior author (C.P.).

### Quality Assessment

The methodological index for nonrandomized studies (MINORS) was used to assess the methodological quality of nonrandomized comparative studies. Each of the 8 questions on the MINORS checklist for noncomparative studies receives a score of 0, 1, or 2, with a maximum score of 16 for noncomparative studies.^9^ Methodological quality was categorized a priori as follows: a score of 0 to 8 or 0 to 12 was considered poor quality, 9 to 12 or 13 to 18 was considered fair quality, and 13 to 16 was considered excellent quality, for comparative and noncomparative studies, respectively. The Cochrane risk-of-bias tool was used to evaluate the quality of randomized trial.^10^ The Cochrane risk-of-bias tool evaluates studies in 7 domains (ie, random sequence generation, allocation concealment, selective reporting, blinding of participants and personnel, blinding of outcome assessment, outcome data, and other biases) as having high, unclear, or low risk of bias.^10^

### Data Abstraction

Two reviewers (N.A., S.S.) independently abstracted relevant data from included articles and recorded the data onto a web-based spreadsheet (Google Sheets, 2021. California: Google LLC) designed a priori. All discrepancies during the extraction phase were resolved after discussion with the senior author (C.P.). Study demographic data included author, year of publication, sample size, level of evidence, and patient demographics for each study. Information regarding the surgical technique, diagnosis that led to the procedure, rehabilitation protocols, postoperative outcomes, and complications were documented. For studies that reported on outcomes of patients undergoing a PRC and in addition had as a subgroup that underwent a PRC + STIA, outcomes of this subgroup were extracted and incorporated when subgroup or individual data points were available.

### Statistical Analysis

Descriptive statistics such as mean, range, and measures of variance (eg, SDs, 95% confidence intervals [CIs]) are presented where applicable. The intraclass correlation coefficient (ICC) was used to evaluate agreement between the reviewers for assessing study quality. A Cohen’s kappa (κ) statistic was used to evaluate inter-reviewer agreement at all screening stages. Agreement was categorized a priori as follows: ICC/κ of 0.81 to 0.99 was considered as almost perfect agreement, ICC/κ of 0.61 to 0.80 was substantial agreement, ICC/κ of 0.41 to 0.60 was moderate agreement, ICC/κ of 0.21 to 0.40 was fair agreement, and ICC/κ of 0.20 or less was slight agreement.^11^ In the title and abstract screening stage, the Cohen’s kappa resulted in a value of 0.24 indicating fair agreement and in the full-text screening stage, a value of 0.46 indicating moderate agreement between the 2 reviewers.

## Results

### Study Characteristics

The search strategy retrieved a total of 1473 articles across the 4 databases. After excluding duplicates, 841 studies were screened and 44 underwent full-text review (Supplemental Figure 1). Eight papers met the inclusion criteria and were included in this evidence synthesis.^4,6,12^-^17^ The included studies were published between 2008 and 2020. Of the studies included in this systematic review, 1 (12.5%) was a cohort study and 6 were case series studies (75%). One randomized controlled trial (RCT) was included in this review (12.5%) (Table 1).^14^

**Table 1.** Study Characteristics.

Author	Total sample size	Sample size (N^a^)	Study design	Level of evidence	% male	Mean follow-up, y (range)	Mean age (range)	MINORS score
Ali et al^12^	61	24	Case series	III	65.4	19.8 (15.1-36.3)	41 (10-72)	11/16
Carneiro et al^13^	14	14	Case series	IV	92.3	1.8 (1-5)	60 (57-73)	12/16
Fukushima et al^14^	30	16	RCT	I	41.9	1	36 (18-54)	—
Gaspar et al^4^	72	25	Cohort study	III	56	5.2 (1.8-11.8)	55.7 (25-81)	11/16
Kwon et al^15^	8	8	Case series	IV	75	3.4 (1.1-3.6)	44.3 (28-64)	12/16
Lee et al^6^	9	9	Case series	IV	77.8	1.5 (1-3)	66.2 (54-74)	11/16
Placzek et al^17^	8	8	Case series	III	—	1	58.5 (50-79)	13/16
Steiner et al^16^	4	4	Case series	IV	75	4.2 (3-5.4)	72.3 (62-78)	12/16

*Note.* RCT = randomized controlled trial; MINORS = methodological index for nonrandomized studies.

a Number of wrists that underwent a proximal row carpectomy with soft tissue interposition.

### Study Quality

Of the studies included in the systematic review, 4 of them were of level IV evidence (50%).^6,13,15,16^ Only 1 study had level I evidence (12.5%).^14^ The remaining 3 studies had level III evidence (37.5%).^1,4,10^ The mean MINORS score for noncomparative studies was 11.7 ± 0.8 (n = 7; 87.5%), respectively (Table 1). One of the studies was an RCT and was assessed using the Cochrane risk-of-bias tool (n = 1; 12.5%) (Table 2).^14^

**Table 2.** Cochrane Risk of Assessment Chart.

Study	Sequence generation	Allocation concealment	Blinding of participants and personnel	Incomplete outcome data	Selective reporting	Other bias
Fukushima et al^14^	No risk	No risk	No risk	High risk	No risk	No risk

### Patient Characteristics and Outcomes

#### Patient demographics

A total of 106 patients (108 wrists) undergoing PRC with STIA were included in this review. The median sample size of patients undergoing a PRC with STI was 11 (range, 4-24). The weighted mean age of all patients was 54.4 ± 12.7 years (ranging from 36 to 66.2 years) (Table 1). The study by Ali et al,^12^ analyzed the demographic data of 24 patients treated with PRC with capsular interposition, but follow-up data were available in only 20 of these patients. The mean follow-up was 4.8 ± 6.3 years (ranging from 1 to 19.8 years) (Table 1). The types of STI used during the PRC surgery were as follows: dorsal capsular interposition (DCI) (n = 80 [74.1%]), acellular dermal allograft scaffold interposition (n = 23 [21.3%]), and meniscal allograft (n = 4 [3.7%]) (Table 3).

**Table 3.** Types of Soft Tissue Interposition Use in Conjunction With Proximal Row Carpectomy.

Study	Sample size (N^a^)	Type of tissue
Ali et al^12^	24	Dorsal capsular interposition
Carneiro et al^13^	14	Acellular dermal allograft
Fukushima et al^14^	16	Dorsal capsular interposition
Gaspar et al^4^	25	Dorsal capsular interposition
Kwon et al^15^	8	Dorsal capsular interposition
Lee et al^6^	9	Acellular dermal allograft
Placzek et al^17^	8	Dorsal capsular interposition
Steiner et al^16^	4	Meniscal allograft

^a^Number of wrists that underwent a proximal row carpectomy with soft tissue interposition.

#### Grip strength

Grip strength was evaluated in 4 studies (50%).^4,6,15,17^ The presentation of this outcome varied within the studies as either an absolute value or a percentage of the contralateral side (Table 4). Three studies (37.5%) provided postoperative information on grip strength in kilograms, with a weighted mean value of 25.4 ± 5.3 kg (n = 25).^6,15,17^ Three studies presented the grip strength as a percentage of the contralateral side, with a weighted mean value of 67.9% (n = 42).^4,6,15^

**Table 4.** Postoperative Grip Strength of Patients Undergoing a Proximal Row Carpectomy With Soft Tissue Interposition.

Study	Sample size (N)	Type of tissue	GS (kg)	GS (%)
Gaspar et al^4^	25	DCI	—	55
Kwon et al^15^	8	DCI	20.3 (10-36.7)	66.8
Lee et al^6^	9	A	30.8 (15.9-49.9)	82
Placzek et al^17^	8	DCI	25	—

*Note.* N = number of wris﻿ts; GS = grip strength; GS (%) = grip strength % of contralateral side; DCI = dorsal capsular interposition; A = dermal allograft.

#### Range of motion

The ROM data were available in 6 of the studies (75%) and are presented in Table 5.^4,6,13,15,16^ Results of ROM at the wrist were presented differently as ROM or total arc of motion in degrees. Postoperative mean ROM of flexion/extension arc was presented in 6 studies (75%), with a weighted postoperative mean of 87.3° ± 15.4° (n = 67; range, 71.9-113).^4,6,13,15,16^ Postoperative information on pronosupination arc was presented in 2 studies (25%), with a weighted mean of 166.3° ± 6° (n = 13; range, 162-170.5).^6,16^ Radioulnar deviation was presented in 2 studies (25%), with a weighted mean of 32.6° ± 5.1° (n = 33, range, 29-36.2).^4,15^

**Table 5.** Postoperative Mean Range of Motion of Proximal Row Carpectomy With Soft Tissue Interposition.

Study	Sample size (N)	Flexion/extension arc (°)	Pronation/supination arc (°)	Radioulnar deviation (°)
Carneiro et al^13^	14	90	—	—
Gaspar et al^4^	25	72	—	29
Kwon et al^15^	8	71.9	—	36.2
Lee et al^6^	9	113	170.5	—
Placzek et al^17^	8	84	—	—
Steiner et al^16^	4	93	162	—

#### Patient-reported outcome measures

Seven of the studies used similar scoring systems to display the patient-reported outcome measures (PROMs) of PRC with STI (Table 6).^4,6,12,14^-^17^ The Disabilities of the Arm, Shoulder, and Hand (DASH) score system was used in 5 of the studies (62.5%). The QuickDASH was used in 1 of the studies. The Patient-Rated Wrist Evaluation (PRWE) scoring system was used in 3 of the studies (37.5%). The weighted mean DASH score was 27.8 ± 8.1 (n = 57; range, 22.3-41.9), and the QuickDASH score was 52 (n = 1), whereas the PRWE score was 41.5 ± 25.9 (n = 3; range, 22.4-31.1).

**Table 6.** Patient-Reported Outcome Measures in Patients Undergoing Proximal Row Carpectomy With Soft Tissue Interposition.

Study	Sample size (N)	Type of soft tissue interposition	DASH score (SD)	QuickDASH score (SD)	PRWE score (SD)
Ali et al^12^	20	DCI	22.3 (7)	—	31.1 (9)
Fukushima et al^14^	16	DCI	41.9 (7.5)	—	—
Gaspar et al^4^	25	DCI	—	10 (14)	71 (19)
Kwon et al^15^	8	DCI	—	—	22.4
Lee et al^6^	9	A	23.8	—	—
Placzek et al^17^	8	DCI	27	—	—
Steiner et al^16^	4	A	24	—	—

*Note.* N = number of wrists; DASH = Disabilities of the Arm, Shoulder, and Hand; PRWE = Patient-Rated Wrist Evaluation; DCI = dorsal capsular interposition; A = allograft (decellularized dermal, acellular dermal, meniscal); SD = standard deviation.

#### Pain

Pain was reported in 6 of the studies (75%).^6,13^-^17^ The Visual Analog Scale (VAS) for pain was used in 3 of the studies (37.5%). The weighted mean postoperative VAS pain score was 3.7 ± 0.6.^13,15,16^ One of the studies used the Cooney system to evaluate pain, and only 1 patient in their study had poor results.^14^ Placzek et al^17^ reported that 2 of the 9 patients failed to acquire any significant pain relief.

#### Patient satisfaction

Regarding patient satisfaction, Gaspar et al^4^ reported that 84% (n = 21) of patients undergoing a PRC with DCI were highly satisfied with the results, and only 2 patients reported feeling neither satisfied nor dissatisfied with the surgery. One of these patients (4%; n = 1) suffered a complication, while the other (4%; n = 1) required conversion to TWA. Lee et al^6^ used a modified Likert scale (5-point questionnaire) for patient satisfaction, reporting a mean postoperative score of 1.5. This scale ranged from 1 to 4, being 1 the highest satisfaction level and 5, the lowest.

#### Complications and reoperations

Complications were report-ed in 3 of the 8 studies (37.5%) (Table 7).^4,14,17^ Fukushima et al^14^ reported 1 patient with superficial site infection of the 16 patients (6.3%) who underwent PRC with DCI. Gaspar et al^4^ reported that 1 of the 24 patients (4.2%) had a deep surgical site infection requiring 2 irrigation and debridement procedures. Reoperations and/or conversion to a TWA were reported in 2 of the studies (25%) (Table 7).^4,14^ Gaspar et al^4^ reported that 1 patient (4%; n = 25) who had PRC with DCI underwent a TWA at 5 years.

**Table 7.** Reported Complications and Reoperations of Patients Undergoing a Proximal Row Carpectomy With Soft Tissue Interposition.

Study	Sample size (N)	Complications (%)	Conversion to TWA (%)
Placzek et al^17^	8	2 (25)	—
Fukushima et al^14^	16	1 (6.3)	—
Gaspar et al^4^	25	1 (4)	1 (4)

*Note.* TWA = total wrist arthrodesis.

## Discussion

The primary finding of this systematic review is that in patients with wrist arthritis involving the lunate fossa and/or proximal pole of the capitate undergoing PRC with STIA, similar clinical results were achieved compared with the ones reported previously in the literature for traditional PRC. Only 5 wrists (10.2%) had complications after PRC with STI, and one (4%) had to be subsequently treated with a revision to a TWA. These findings suggest that PRC with STIA can be a viable alternative for patients with degenerative changes at the lunate fossa or capitate.

Some studies included in this review mentioned that the use of an STIA in conjunction with PRC had similar results when compared with a PRC alone.^4,14,16^ Kwon et al^15^ reported significant improvements in the preoperative versus the postoperative PRWE scores with a mean change of 44.4. The only RCT included in this review, which analyzed outcomes of 16 patients, reported that there was no difference in the outcomes (DASH score, Cooney’s system, and complications) of patients with PRC only and PRC with DCI.^14^ The results of this study failed to show differences in wrist function or in complication rate after these procedures. Gaspar et al^4^ recorded improvements in PRWE scores with the use of capsular interposition when compared with traditional PRC, with a mean lower change of 44 in PRWE scores as opposed to 41, respectively. The use of interposition can be beneficial to patients suffering from degenerative changes at the capitate proximal pole and lunate fossa.^4^ In addition, postoperative PROMs presented for patients undergoing PRC with STIA showed moderate disability. The weighted mean DASH score was 27.8 ± 8.1 (n = 57; range, 22.3-41.9), and the QuickDASH score was 52 (n = 1), whereas the PRWE score was 41.5 ± 25.9 (n = 3; range, 22.4-31.1). Based on the interpretation of the scores, the DASH scores show moderate disability in patients. The QuickDASH score shown in Gaspar et al^4^ also demonstrated moderate disability.

Scores within the 0 to 29 range on the DASH scale suggest the absence of substantial upper limb disorders in patients.^16^ Of the studies that included DASH outcomes, in only 2 studies (50%; n = 2), postoperative DASH scores were more than 29 (DASH score values of 41.9 and 52, respectively).^4,14^ However, in the study by Ali et al,^12^ although DASH scores were higher than 29, outcomes in the PRWE and DASH scores showed no difference when comparing PRC with DCI with only PRC. The average PRWE and DASH scores were 37.2 ± 10 and 25.2 ± 9, respectively. However, patients with capsular interposition (32.8%; n = 20) showed scores of 31.1 ± 9 and 22.3 ± 7.

In advanced cases of arthritis, TWA is considered as one of the main treatment options.^4^ Even though this procedure can reliably treat the patients’ pain, it has the great disadvantage of completely sacrificing wrist motion.^4,15^ Furthermore, it still has a higher complication rate when compared with PRC (29% vs 14%, respectively).^4^ Therefore, in light of our findings, adding a STIA is an attractive alternative. This must be planned, and it only adds few minutes to the operating time; the capsulotomy must be performed in a distally based flap if autologous tissue is desired as the interposition tissue.^18^

### Limitations

The main limitation of this review relies on the small number of studies and their low quality. Unfortunately, PRC with STIA has not been extensively studied in the literature. The literature on outcomes of PRC with STI consisted mostly of retrospective cohort studies using a wide variety of clinical scoring systems and the availability of data distinguishing between PRC and PRC with interposition arthroplasty is limited. Furthermore, some studies failed to differentiate the score results of PRC versus PRC with STIA, which impaired our ability to include additional patients and impacted our sample size.^6,12,13,16,17^ Failures, complications, and reoperation rates in studies were mentioned, but some studies did not report whether the complications occurred to the PRC with STIA group or not. It should also be noted that the sample size in each of the studies with PRC with STIA was small.

Finally, given the lack of standardization in arthritis severity reporting, there was no detailed information regarding the location and extent of the cartilage damage of the capitate proximal pole and/or lunate fossa of the radius which makes the external validity and applicability of these findings challenging.

### Future Directions

Further prospective studies are needed to be able to decide on which are the best indications for PRC with STI and compare long-term outcomes of PRC versus PRC with STI procedures, while analyzing the difference in the type of STI used. In addition, studies comparing the results of patients undergoing PRC with STIA and alternative procedures, such as PRC with capitate resurfacing arthroplasty, are needed. A study by Rahgozar et al^19^ evaluated the rates of TWA conversion and total cost in patients who underwent PRC. The results indicated that there was an increased rate of TWA conversion in patients who underwent 4-CF compared with PRC, (19.2%-4.9%; *P* < .001) as well as a greater average cost ($10 842 vs $7171; *P* < .01). Furthermore, studies discussing salvage wrist procedures such as PRC should use standardized surgical outcome scoring systems and PROMs. Developing and using a core outcome set would be beneficial to design future studies in this population. Finally, further scientific research into the impact of STIA on wrist kinematics is required.

## Conclusions

For patients with painful wrist arthritic conditions who have lunate fossa and/or capitate involvement, the use of PRC with STIA seems to have satisfactory short-term and mid-term outcomes, comparable with conventional PRC. Furthermore, complications are either similar or lower than other wrist salvage procedures such as PRC alone, 4-CF, TWA, or wrist arthroplasty.^18^ Future higher quality prospective studies are needed to assess the outcomes after a PRC with STI with mid-term and long-term follow-up. The use of a core outcome set for these patients can be beneficial to compare different studies to conclude whether PRC with STI is an advantageous option for wrist arthritis.

## Supplemental Material

sj-docx-1-han-10.1177_15589447231221245 – Supplemental material for Proximal Row Carpectomy With Soft Tissue Interposition: A Systematic Review of Clinical Outcomes Supplemental material, sj-docx-1-han-10.1177_15589447231221245 for Proximal Row Carpectomy With Soft Tissue Interposition: A Systematic Review of Clinical Outcomes by Neveadan Aravinthan, Salwa Siddiqui, Moin Khan, Jaydeep Moro, Paula A. Pino and Carlos Prada in HAND

## Appendix

### Search Terms

1. Proximal Row Carpectomy
2. Surgery
3. Surgical Procedures
4. Surg*ery*
5. Outcomes
6. Wrist Arthritis
7. Or/ 1-4
8. and/ 1-6
9. Wrist joint*
10. Wrist joint/ surgery
11. Carpal bones/ surgery*
12. osteoarthritis/ surgery/ OA
13. Scaphoid bone/ surgery*
14. Soft tissue interposition
15. Carpal Joints (Mesh)
16. Tissue interposition
17. allograft

### PubMed

- proximal row carpectomy and surgery and complications and carpal joints (12 results)
- (((proximal row carpectomy) AND (surgery)) AND (wrist joint*)) AND (complications) (93 results)
- Proximal Row Carpectomy.ab AND surg* AND complications (208 results)
- ((carpal joint) AND (complication*)) AND (proximal row carpectomy/) (84 results)
- proximal row carpectomy and surgery and wrist joint and osteoarthritis (86 results)
- proximal row carpectomy or soft tissue interposition and surgery and carpal joint and complications and wrist arthritis (372 results)
- proximal row carpectomy and tissue interposition (11 results)
- proximal row carpectomy and allograft (6 results)
- Proximal Row Carpectomy.ab AND (surg* OR complications) (704 results)
- Proximal Row Carpectomy.ab AND (surg* OR complications OR soft tissue OR graft) (711 results)

### Cochrane

- Surgical procedures and complications or carpal joints
- proximal row carpectomy and surgery and wrist joint and osteoarthritis (3 results)
- Proximal Row Carpectomy (8 results)
