# Supplementary figures and images for: Proximal Row Carpectomy With Soft Tissue Interposition: A Systematic Review of Clinical Outcomes
Source: Hand (N Y). 2024 Jan 30;20(3):352–9. doi: 10.1177/15589447231221245 (PMC11571415; doi:10.1177/15589447231221245)

**Supplementary Figure 1.** PRISMA flow chart


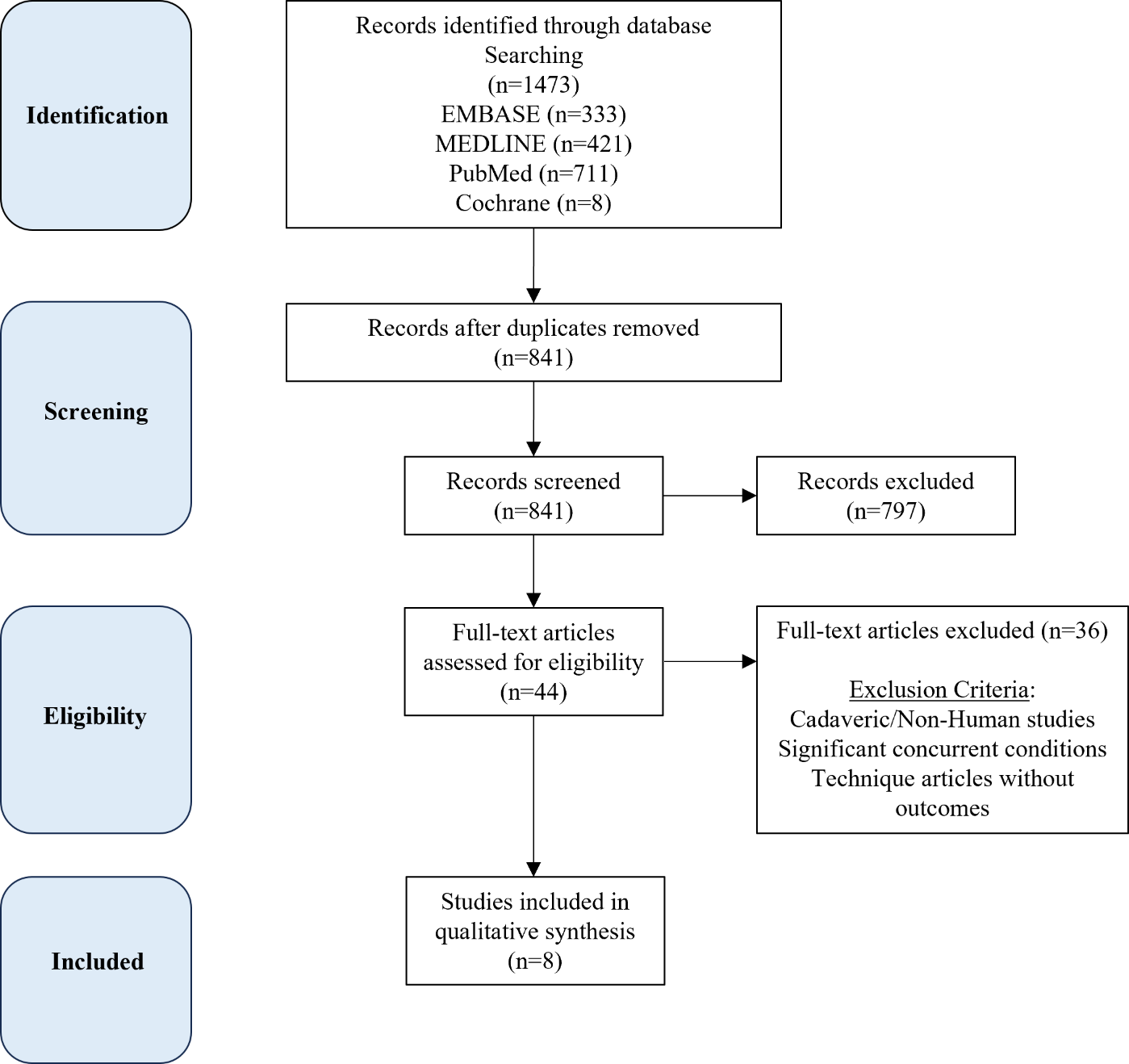

Supplement: sj-docx-1-han-10.1177_15589447231221245 – Supplemental material for Proximal Row Carpectomy With Soft Tissue Interposition: A Systematic Review of Clinical Outcomes [file sj-docx-1-han-10.1177_15589447231221245.docx]
